# Pre-K–12 Teachers’ Views on ASD+ADHD: Prevalence Estimates and Teaching Preparedness

**DOI:** 10.3390/children12030342

**Published:** 2025-03-09

**Authors:** Sidni A. Justus, Emily M. Pogue, Victoria Simanovich

**Affiliations:** 1 Department of Psychological Science, Kennesaw State University, Kennesaw, GA 30144, USA; vsimanov@students.kennesaw.edu; 2 College of Science and Mathematics, Kennesaw State University, Kennesaw, GA 30144, USA; epogue@students.kennesaw.edu

**Keywords:** ASD, ADHD, neurodevelopmental disorders, comorbidity, neurodiversity, inclusive education, teacher preparedness

## Abstract

Background/Objectives: Teachers play a significant role in the identification and intervention of neurodevelopmental disorders such as ASD and ADHD. However, their perceptions of ASD+ADHD comorbidity remain underexplored. This study investigates teachers’ estimates of ASD+ADHD prevalence and their perceived preparedness to teach students with co-occurring diagnoses, exploring key predictors of both outcomes. Methods: Pre-K-12 teachers (*N* = 199) completed demographic questions and four additional questionnaires assessing ASD- and ADHD-specific knowledge, neurodiversity attitudes and overall teaching self-efficacy. Participants estimated the prevalence of ASD+ADHD comorbidity and rated their preparedness to instruct students with ASD+ADHD, ASD-only, and ADHD-only. Regression analyses examined factors predicting prevalence estimates and preparedness. Results: Teachers underestimated the lifetime prevalence of ASD+ADHD, though current prevalence estimates were more aligned with meta-analytic findings. Personal experience and current teaching of comorbid students predicted higher prevalence estimates, whereas greater ASD knowledge was associated with lower estimates. Preparedness to teach ASD+ADHD was rated lower than for ASD-only or ADHD-only students. Self-efficacy, neurodiversity attitudes, and professional training were predictors of preparedness. Current teaching of comorbid students, disorder-specific knowledge, and total years of teaching experience showed inconsistent associations across conditions. Conclusions: Findings suggest that while direct experience and training enhance both awareness and preparedness, disorder-specific knowledge alone does not consistently translate to confidence in supporting students with co-occurring diagnoses. Implications for teacher training and the need for comorbidity-focused professional development are discussed.

## 1. Introduction

Autism Spectrum Disorder (ASD) and Attention-Deficit/Hyperactivity Disorder (ADHD) are among the most common neurodevelopmental disorders in school-aged children [1]. ADHD is primarily characterized by severe deficits in attention, hyperactivity, and impulsivity, whereas ASD is associated with impaired communication and social interaction skills, as well as restricted and repetitive behaviors [2]. Despite these seemingly distinct diagnostic criteria, ASD and ADHD frequently co-occur. In the present study, we use the term ASD+ADHD to refer to this comorbid presentation. ASD+ADHD prevalence estimates vary widely (e.g., 40–70%) depending on methodological approaches, sample characteristics, and whether ASD or ADHD is the primary diagnosis [3]. Recent meta-analytic findings suggest that approximately 38–40% of individuals with ASD also meet criteria for ADHD [4], with age-related variability in comorbidity rates ranging from 18% in children under six to 48% in adolescents. However, large-scale U.S. administrative data report considerably lower prevalence, with rates as low as 1.2% [5]. These discrepancies may further stem from differences in diagnostic frameworks over time. ASD and ADHD were historically viewed as mutually exclusive diagnoses, and until DSM-5 [6], a dual diagnosis was not permitted. This restriction likely contributed to underdiagnosis and misclassification, delaying appropriate intervention and educational support [7,8].

Compounding these challenges, many children with ASD+ADHD may still experience delayed or sequential diagnoses. ADHD symptoms, particularly hyperactivity and impulsivity, often emerge first, resulting in an initial ADHD diagnosis before ASD is identified [9,10,11]. Studies suggest that approximately half of children who received a late ASD diagnosis were previously diagnosed with another neurodevelopmental disorder, most commonly ADHD [9,12]. Furthermore, children with co-occurring ADHD receive their ASD diagnosis an average of one to three years later than peers with ASD alone [10,11], delaying access to specialized intervention services [13]. This pattern aligns with findings that ASD is typically diagnosed later when ADHD is present. At the same time, ADHD is identified earlier when ASD is present, suggesting that the presence of one condition may alter the diagnostic trajectory of the other [14]. These diagnostic delays can have significant implications for educational settings, leading to mismatched instructional strategies, inappropriate behavioral attributions, and challenges in securing appropriate accommodations. While research has indicated that 69% of students with ADHD [15] and 82% or more of students with ASD [16] receive academic accommodations and support, little is known about how well educators understand and address the distinct needs of students with ASD+ADHD.

### 1.1. The Role of Teachers in Supporting Neurodivergent Students

Teachers are often among the first professionals to recognize neurodevelopmental differences in children [17,18,19], playing a key role in identifying student needs, implementing classroom interventions, and securing necessary accommodations. However, research on teacher accuracy in identifying ASD and ADHD is mixed. Some have found that teachers can recognize core ASD traits, e.g., [20]. However, a recent meta-analysis indicates that teachers possess limited ASD knowledge [21]. Teacher recognition of ADHD symptoms is similarly debated [22,23]. While teachers are not trained clinicians, their ability to recognize and differentiate between ASD and ADHD symptoms accurately is critical for timely diagnosis and intervention, given that children spend a significant portion of their time in school.

Research indicates that teachers’ ability to recognize ASD and ADHD symptoms with increased experience with the disorder and targeted training [21,24,25,26]. However, despite these benefits, many teachers report feeling underprepared to support neurodivergent students [27,28]. The Individuals with Disabilities Education Act (IDEA) of 2004 [29] emphasizes including students with disabilities in general education classrooms. However, general education teachers often receive minimal formal training in special education, leaving them underprepared for this responsibility. Rosenzweig [30] found that general education teachers typically take only 1.5 courses on inclusion or special education during their training, compared to special education teachers, who take an average of 11 such courses. Conversely, special education teachers’ preparation for inclusive general education settings is often limited as well [31]. This disparity suggests that neither training sequence fully equips teachers to effectively support students with neurodiverse diagnoses, a challenge that becomes even more complex in the presence of comorbidity.

These training gaps directly influence teachers’ preparedness to support neurodivergent students. Preparedness is closely tied to teachers’ sense of self-efficacy or judgment of their ability to bring about desired student outcomes through their teaching [32]. Bandura suggests that teaching self-efficacy is influenced by direct experiences, vicarious experiences (e.g., watching others teach/interact with neurodivergent students), and perceived stress or physiological reactions to challenges [32]. In educational contexts, teaching self-efficacy has been conceptualized in different ways. For example, some distinguish between two factors: generalized (i.e., individuals’ expectation that teaching can influence student learning) and personal self-efficacy (i.e., individuals belief that they themselves have skills to bring about that learning) [33]. Others have suggested that self-efficacy be domain-specific, with variations across instructional strategies, classroom management, and student engagement [34]. However, little research has explored whether general teaching self-efficacy translates to explicit self-reported preparedness for supporting neurodivergent students, particularly those with comorbid presentations.

Beyond knowledge, training, and self-efficacy, teachers’ attitudes toward neurodiversity also significantly impact their effectiveness in inclusive classrooms [35,36]. Negative attitudes or misconceptions about neurodevelopmental conditions can hinder the implementation of inclusive practices [37]. As with knowledge training, studies have also demonstrated that training focused on neurodiversity and inclusive design can reduce negative thinking and promote inclusive teaching practices [38,39]. However, little research has examined how well teachers recognize ASD+ADHD as a distinct comorbid condition or whether their experiences, knowledge, and attitudes influence their understanding of its prevalence and their confidence in supporting affected students.

### 1.2. The Present Study

Despite growing conversations about ASD+ADHD, research has explored how teachers conceptualize the co-occurrence of these conditions or whether their estimates align with empirical data. Additionally, while prior research has examined teacher preparedness to support students with ASD or ADHD independently, to our knowledge, no studies have explicitly investigated whether teachers feel adequately prepared to instruct students with both conditions. Given the increasing push for inclusive education, understanding teachers’ ASD+ADHD prevalence estimates and preparedness is a crucial first step for improving teacher training and classroom support.

To address these gaps, the present study examines two key research questions. First, (RQ1) we investigate how teachers estimate the prevalence of ASD+ADHD comorbidity and whether these estimates are associated with their teaching or personal experience, prior training, disorder-specific knowledge, and general attitudes toward neurodiversity. Second (RQ2), we assess teachers’ self-reported preparedness to instruct students with ASD+ADHD, evaluating whether their perceived preparedness is influenced by similar factors. Additionally, we compare their preparedness for teaching students with comorbid ASD+ADHD to ratings of preparedness for teaching students with only ASD or only ADHD.

For RQ1, we hypothesized that teachers with increased experience with ASD+ADHD (teaching and personal) would provide higher estimates of comorbidity, as firsthand exposure may increase awareness of cooccurrence. Similarly, more positive attitudes towards neurodiversity could be associated with higher estimates if those with greater acceptance of neurodiversity also view comorbid conditions as more prevalent. However, we did not make a priori predictions for disorder-specific knowledge and prior training, as these factors could plausibly increase or decrease estimates. Higher ASD- and ADHD- specific knowledge might lead to higher estimates if greater familiarity with both conditions promotes recognition of their overlap. Alternatively, greater knowledge might lead to lower estimates if teachers conceptualize each as a distinct condition rather than one that frequently co-occurs with the other.

For RQ2, we hypothesized that all predictors (greater experience, disorder-specific knowledge, training, neurodiversity attitudes) in addition to overall teaching self-efficacy would predict greater perceived preparedness to teach students with ASD+ADHD. Finally, we hypothesized that teachers would report feeling less prepared to teach students with ASD+ADHD compared to those with only ASD or ADHD.

## 2. Materials and Methods

### 2.1. Participants

Participants were recruited through Prolific (www.prolific.com), an online research recruitment platform that allows researchers to pre-screen participants based on specific demographic and professional criteria. Prolific was chosen for this study because it allowed for targeted recruitment of teachers, advertising only to individuals who had previously indicated teaching as their profession in their Prolific demographic profile. Compared to other online platforms, Prolific has been shown to yield higher quality data (e.g., greater participant attentiveness and fewer invalid responses), making it a reliable tool for behavioral and educational research [40].

Final eligibility was confirmed within the survey, requiring participants to be currently employed as teachers (preschool–high school) and to have experience teaching at least one of the following groups: (1) students with ASD, (2) students with ADHD, or (3) students diagnosed with both ASD and ADHD. A total of 215 participants completed this study. Fourteen participants were excluded from analysis due to eligibility concerns (*n* = 1 not currently employed as a teacher; *n* = 13 did not report current experience teaching students with ASD, ADHD, or comorbid diagnoses). Two additional participants were excluded due to self-reported concerns about potential response errors on a section of the survey that could not be verified. This resulted in a final analytic sample of 199 teachers. Although the minimum age in our sample was 18, all individuals aged 18–19 (*n* = 4) were teaching in early childhood education settings, likely in private daycare or pre-kindergarten programs where formal teaching certification is not always required. Their inclusion aligned with our eligibility criteria, as all participants were actively teaching neurodivergent students (ASD, ADHD, and/or ASD+ADHD).

The final sample of participants ranged in age from 18 to 65 years (*M* = 39.55, *SD* = 10.77). The majority (74.9%) identified as female, with 24.6% identifying as male and 0.5% identifying as gender non-conforming or other. The racial composition of the sample was 85.4% White, 6.5% Black or African American, 1% Asian, 1% American Indian or Alaska Native, 2% Multi-racial, and 4% Other. Additionally, 5% of participants identified as Hispanic or Latino ethnicity, which was separately reported from race. The highest level of education completed varied with 55.8% holding a master’s degree, 36.2% a bachelor’s degree, 3% an associate’s degree, 3% some postsecondary education, and 2% a doctoral degree.

#### Teaching-Related Characteristics

In addition to general demographics, participants provided additional teaching-related background information, including primary teaching role (e.g., general education classroom teacher, special education teacher), school type, school setting, the socioeconomic background of students served, and the level of classroom integration for neurodivergent students (summarized in Table 1). Participants ranged from less than 1 to 47 years of teaching experience (*M* = 12.57, *SD* = 8.58) and the majority (92.5%) were currently employed full-time.

### 2.2. Measures

#### 2.2.1. Participatory Autism Knowledge Measure (PAK-M)

The PAK-M was used to measure teachers’ ASD-specific knowledge. The PAK-M was originally developed as a 13-item scale by Stone and colleagues [41,42] and recently updated by Gillespie et al. [43] to reflect updated diagnostic criteria and add items that also focus on adult-related outcomes (e.g., “Autistic people can lead successful and satisfying lives”). The updated 29-item measure was used in the present study. Participants were asked to indicate agreement with each statement using a 5-point (“Strongly Agree” to “Strongly Disagree”) Likert-type scale with scoring ranging from −2 to 2 for each item. Scores are summed (range: −58–58), where higher scores indicate more knowledge regarding ASD. Cronbach’s alpha for the PAK-M in this study was α = 0.92.

#### 2.2.2. Scale of ADHD-Specific Knowledge (SASK)

The SASK was used to measure teachers’ ADHD-specific knowledge. The SASK is a 20-item measure (e.g., “ADHD is a neurobiological, developmental disorder”) from the larger ADHD-Specific Knowledge and Attitudes of Teachers (ASKAT) [44] instrument, which assesses both knowledge and attitudes toward ADHD. In the present study, only the knowledge subscale was included to parallel the PAK-M, which measures autism-specific knowledge. Although the original SASK items were designed as true/false questions, the present study mirrored the approach used in the Neurodiversity Attitudes Questionnaire (NDAQ; see Section 2.2.3) validation study [45] by using the 5-point (“Strongly Agree” to “Strongly Disagree”) Likert-type scale and −2 to 2 scoring system from the PAK-M. This adaptation aligned the response format across both ASD- and ADHD-specific knowledge measures, facilitating direct comparisons between participants’ knowledge of each condition. Cronbach’s alpha for the SASK in this study was α = 0.80.

#### 2.2.3. Neurodiversity Attitudes Questionnaire (NDAQ)

The NDAQ [45] was used to measure teachers’ overall attitudes toward neurodiversity. The NDAQ was initially validated in a sample of individuals currently working in or intending to work in helping professions (“…broadly defined as anyone involved in education, medicine or other therapy” [5] (pp. 2824–2825). The measure includes 28 items measuring five factors, including Diversity and Inclusion (8 items; e.g., “There is no such thing as a “normal” brain.”), Fitting in (4 items; e.g., “Neurodivergent people should learn social skills in order to fit in with their peers.”), Cross-Neurotype Interactions (5 items; e.g., “I would be friends with a neurodivergent person.”), Medical Model (6 items, e.g., “I support organizations that want to find a cure for autism.”), and Listening and Reflecting (5 items, e.g., “I spend time thinking about how to make things more sensory-friendly for neurodivergent people”). Instructions first inform participants that the term neurodiversity represents certain conditions, including autism, ADHD, intellectual disabilities, dyslexia, dyspraxia, Tourette’s, and other cognitive differences. Participants are then asked to rate their level of agreement with each statement using a Likert-type scale from ”1 = Strongly Disagree” to “9 = Strongly Agree”. Although the NDAQ was designed to assess distinct dimensions of neurodiversity attitudes, the validation study also presents a total score (range: 28–140) as an overall index of neurodiversity-related beliefs. The total score previously demonstrated convergent validity with other measures included in this study, including the Participatory Autism Knowledge Measure (PAK-M) and the Scale of ADHD-Specific Knowledge (SASK) [45]. The present study used the total NDAQ score, with higher scores reflecting more positive attitudes toward neurodiversity. Cronbach’s alpha for the NDAQ in this study was α = 0.91.

#### 2.2.4. Teacher Sense of Self Efficacy Scale (TSES)

The Short-Form of the TSES [34] was used to measure teachers’ overall evaluations of their general teaching confidence and likelihood of success. The Short-Form of the TSES includes 12 items measuring three subscales (four items each) of teacher self-efficacy in student engagement (e.g., “How much can you do to motivate students who show low interest in school work?”), instructional strategies (e.g., “How much can you use a variety of assessment strategies?”), and classroom management (e.g., “How much can you do to control disruptive behavior in the classroom?”). Participants are asked to respond by giving their opinion on each statement using a Likert-type scale from “1 = Nothing” to “9 = A Great Deal”. Scores for each subscale (range: 4–36) and the total (range: 12–108) are summed. The present study used the total TSES score, with higher scores reflecting more positive attitudes towards higher scores indicating greater self-efficacy. Cronbach’s alpha for the TSES in this study was α = 0.90.

#### 2.2.5. Teacher Preparedness

Teacher preparedness was assessed using a single-item measure for each diagnostic category: ASD, ADHD, and ASD+ADHD. Participants were asked, “How prepared do you feel to support students with each of the following diagnoses?” and responded on a Likert-type scale from “1 = Not at all Prepared” to “5 = Extremely Prepared”. Higher scores indicated greater self-reported confidence in supporting students with the respective condition. Unlike general teaching self-efficacy (TSES), which measures broad instructional confidence, this additional measure specifically assessed teachers’ perceived ability to support neurodivergent students.

### 2.3. Procedure

All questionnaires and tasks for this study were administered via Qualtrics [46], a secure online survey platform commonly used in academic research and hosted for online data collection through Prolific [47]. Participants were informed of the study procedures and inclusion criteria on the Prolific advertisement and informed consent form. Electronic consent form was obtained via Qualtrics before participation. Participants then completed screening questions to confirm that they were currently employed as teachers and were teaching students diagnosed with one or more of the following: ASD, ADHD, or co-occurring ASD and ADHD.

Eligible participants then proceeded to complete self-report questions regarding teaching-related background characteristics, followed by the four questionnaires (PAK-M, SASK, NDAQ, TSES), ratings of preparedness to teach students with each diagnosis (ASD, ADHD, ASD+ADHD), and general demographic questions. Periodic attention checks (i.e., nonsensical questions with a specific correct answer) were embedded throughout the study to ensure data integrity. Participants included in the final analysis for this study passed all attention checks. All study components were completed in a fixed order during a single online session lasting approximately 30 min. Participants were compensated USD 5 (equivalent to USD 10/h) in accordance with Prolific’s compensation policies.

### 2.4. Analysis

All statistical analyses were performed using IBM SPSS 29 software [48]. Two multiple regression analyses were performed to examine predictors of teachers’ estimated prevalence of ASD+ADHD comorbidity (RQ1) and perceived preparedness to support students with ASD+ADHD (RQ2). Predictors included background factors (i.e., total years of teaching experience, personal experience (self/family diagnosis) with ASD, ADHD, or ASD+ADHD), exposure and experience (i.e., current teaching off students with comorbid ASD+ADHD, prior training in teaching ASD, ADHD, and/or ASD+ADHD) and knowledge and attitudes (i.e., ASD knowledge, ADHD knowledge, and neurodiversity attitudes). Given that preparedness reflects teachers’ confidence in their ability to effectively support students with ASD, ADHD, and co-occurring ASD+ADHD, the Teacher Sense of Efficacy Scale (TSES) was included as an additional predictor in the regressions for RQ2. Self-efficacy was not examined in RQ1, as comorbidity prevalence estimates are more likely influenced by knowledge and exposure than personal teaching confidence.

Given the wide age range in our sample (18–65 years), we examined whether age contained extreme outliers using interquartile range (IQR) analysis and boxplot inspection. No extreme outliers were identified, and age was retained in descriptive analyses. We also explored whether age was related to predictors of interest and found that age and years of teaching experience were strongly correlated (*r* = 0.726, *p* < 0.001), meaning older participants generally had more experience. Since professional exposure is more relevant to preparedness than age alone, we used the total years of teaching experience as the predictor in regression models rather than age. To assess the robustness of our findings, we also conducted a sensitivity analysis excluding the four youngest participants (ages 18–19) to determine whether their inclusion influenced results. The exclusion of these participants did not change any significant effects, supporting their retention in the final analyses.

Prior to analysis, we also evaluated assumptions of normality (Shapiro–Wilk’s test *p* < 0.05 and inspection of Q-Q plots), linearity (scatterplots), and homoscedasticity (residual plots). For regressions, we confirmed no multicollinearity concerns (Variance Inflation Factors < 2.0). For repeated measures, Analysis of Variance (ANOVA; RQ2), Mauchly’s test indicated a violation of sphericity, and Greenhouse–Geisser corrections were applied. Where *t*-tests were used, effect sizes were calculated using Cohen’s *d*, where *d* = 0.2 is considered a small effect, *d* = 0.5 is considered a medium effect, and *d* = 0.8 is considered a large effect size [49]. For regressions, adjusted R^2^ was used as the estimate of model fit. For ANOVA, effect sizes were calculated using partial eta-squared η*p*^2^, where η*p*^2^ = 0.01 is considered a small effect, η*p*^2^ = 0.06 is considered a medium effect, and η*p*^2^ = 0.14 is considered a large effect size [49].

## 3. Results

### 3.1. Teachers’ Estimates of ASD+ADHD Comorbidity (RQ1)

Descriptive statistics indicated that teachers’ estimated prevalence of co-occurring ASD and ADHD ranged from 0% to 88% (*M* = 36.56%, *SD* = 20.66). To determine whether these estimates aligned with empirical estimates of ASD+ADHD comorbidity, one-sample *t*-tests were conducted comparing teachers’ estimates to 38.5% (current prevalence) and 40.2% (lifetime prevalence) benchmarks as reported in a meta-analysis by Rong et al. [4]. The results revealed that the teachers’ estimates did not significantly differ from the current prevalence of 38.5%, *t*(198) = −1.33, *p* = 0.093, and Cohen’s *d* = 0.09. However, estimates were significantly lower than the lifetime prevalence of 40.2%, *t*(198) = −2.49, *p* = 0.007, and *d* = 0.18. These findings suggest that while teachers’ estimates approximate the lower-end empirical estimates, they may underestimate the persistence of ASD+ADHD over time. Given that meta-analytic research has shown that ASD+ADHD comorbidity estimates also vary by age group [4], a follow-up analysis was conducted to explore whether teachers’ estimates differed based on the grade levels they currently taught. A one-way ANOVA comparing prevalence estimates across four teaching levels (Pre-K/K, elementary, middle, and high) indicated no significant differences across groups, *F*(3,195) = 1.41, *p* = 0.243, ηp² = 0.02. These findings suggest that teachers’ perceptions of ASD+ADHD prevalence remain relatively stable regardless of the age group they teach.

To examine factors that may predict teachers’ estimations of ASD+ADHD comorbidity, an exploratory multiple regression analysis was conducted (Table 2). Among the predictors, current teaching experience with students with comorbid ASD+ADHD was significantly associated with higher estimates, as was personal experience with ASD, ADHD, or comorbid diagnoses (self-and/or family member). These results suggest that teachers with direct exposure, either through their students or personal connections, tended to estimate higher comorbidity prevalence rates. Conversely, total years of teaching experience was associated with lower comorbidity estimates, suggesting that more experienced teachers tended to provide lower estimates of ASD+ADHD co-occurrence. Greater ASD knowledge (PAK-M) was also negatively associated with comorbidity estimates, indicating that teachers with more ASD-specific knowledge provided lower estimates of ASD+ADHD co-occurrence. ADHD knowledge (SASK) and attitudes towards neurodiversity (NDAQ) were not significant predictors (*p* > 0.05).

### 3.2. Perceived Preparedness Across Diagnoses (RQ2)

Descriptive statistics indicated that teachers generally felt moderately prepared to teach students with ASD (*M* = 3.67, *SD* = 0.97), ADHD (*M* = 3.93, *SD* = 0.88), and co-occurring ASD+ADHD (*M* = 3.29, *SD* = 1.09). A repeated-measures ANOVA was conducted to examine whether perceived preparedness differed across ASD, ADHD, and co-occurring ASD+ADHD. Mauchly’s test indicated that the assumption of sphericity was violated, χ^2^(2) = 14.03, *p* < 0.001; therefore, Greenhouse–Geisser correction was applied. The results revealed a significant main effect of diagnosis, *F*(1.87, 370.52) = 58.49, *p* < 0.001, η*p*^2^ = 0.23, indicating that preparedness ratings differed significantly across conditions. Post hoc comparisons indicated that teachers felt more prepared to support students with ADHD than ASD: *t*(198) = 3.998, *p* < 0.001, and Cohen’s *d* = 0.28. Similarly, teachers felt more prepared to support students with ASD compared to co-occurring ASD+ADHD: *t*(198) = 7.47, *p* < 0.001, and Cohen’s *d* = 0.53. Finally, teachers reported feeling most prepared to support students with ADHD relative to those with co-occurring ASD+ADHD: *t*(198) = 10.28, *p* < 0.001, and Cohen’s *d* = 0.73. Taken together, these findings suggest that teachers perceive greater difficulty supporting students with comorbid ASD+ADHD compared to either condition independently.

To examine factors predicting teachers’ preparedness to support students with co-occurring ASD+ADHD, a multiple regression analysis was conducted (Table 3). Predictors for RQ2 were the same as RQ1 with the addition of teaching self-efficacy (TSES), given its theoretical relevance to teacher preparedness. Among the predictors, current teaching experience with students with ASD+ADHD was significantly associated with greater perceived preparedness to teach these students, as was formal training related to either ASD, ADHD, and/or comorbid ASD+ADHD. These results suggest that direct, hands-on experience and structured training opportunities are critical in fostering teachers’ confidence in supporting students with co-occurring diagnoses. Additionally, more positive attitudes toward neurodiversity and higher general teaching self-efficacy were associated with greater perceived preparedness, indicating that teachers who embrace neurodiversity and feel confident in their overall teaching abilities may also feel more prepared to support students with co-occurring diagnoses. However, ASD- and ADHD-specific knowledge scores were not significant predictors, nor were total years of teaching experience or personal experience with neurodevelopmental conditions (*p* > 0.05).

For comparison, separate regression analyses were conducted examining predictors of preparedness for teaching students with only ASD and only ADHD (see Supplementary Tables S1 and S2). Notably, the pattern of significant predictors varied slightly across models. For preparedness to teach students with ASD, formal training (*p* < 0.001), neurodiversity attitudes (*p* < 0.001), and general teaching self-efficacy (*p* = 0.001) were significant predictors, consistent with the ASD+ADHD model. Similarly, ASD- and ADHD-specific knowledge scores were again not significant predictors (*p* > 0.05), nor was personal experience with neurodevelopmental conditions or total years of teaching experience. Current teaching experience with students with ASD+ADHD was not a significant predictor of preparedness to teach ASD-only students (*p* = 0.105). This suggests that while direct experience with students who have both ASD and ADHD contributes to confidence in supporting comorbid presentations, it does not necessarily translate to increased preparedness for ASD-specific instructions.

For preparedness to teach students with ADHD, formal training (*p* = 0.022), neurodiversity attitudes (*p* = 0.005), and general teaching self-efficacy (*p* < 0.001) remained significant predictors, mirroring the ASD and ASD+ADHD models. ADHD-specific knowledge, total years teaching, and personal experience were not significant predictors, also mirroring the other two models. Unlike the other models, greater ASD-specific knowledge (*p* = 0.020) emerged as a predictor of lower perceived preparedness to teach students with ADHD, suggesting that a stronger understanding of ASD does not necessarily translate into confidence in supporting students with ADHD.

## 4. Discussion

Teachers play a central role in recognizing and supporting neurodivergent students, who are increasingly being integrated into mainstream classrooms. While prior research has examined teachers’ knowledge and attitudes toward specific conditions, including ASD and ADHD, few studies have investigated how they perceive the comorbid overlap of these conditions. If teachers lack awareness of ASD+ADHD comorbidity or feel ill-equipped to address its unique challenges, training programs may need to shift toward more integrated approaches that account for co-occurring needs. The present study examined teachers’ estimates of ASD+ADHD prevalence and their self-reported preparedness to instruct students with co-occurring as well as separate diagnoses, identifying key factors that influence both. Below, we discuss findings in relation to our research questions, highlighting implications for teacher training and student support.

### 4.1. Estimating ASD+ADHD Comorbidity

Teachers significantly underestimated the lifetime prevalence of ASD+ADHD comorbidity, while their estimates of current prevalence were more aligned with meta-analytic findings [4]. This discrepancy highlights a potential gap between empirical data and educators’ perceptions. Given the variability in reported comorbidity rates across studies [3,4,5], it is unsurprising that teachers’ estimates differed from these rates to some degree. However, the underestimation of lifetime prevalence compared to current prevalence may suggest a lack of awareness regarding the persistence of ASD+ADHD across development. Since ADHD symptoms can become less overt with age and intervention [50,51,52], teachers may assume that co-occurring diagnoses become less common over time, even though empirical evidence suggests otherwise [4]. Future research, perhaps utilizing qualitative methodology (e.g., semi-structured interviews), could explore whether this discrepancy reflects a genuine lack of knowledge about long-term comorbidity patterns or if it is primarily driven by classroom experiences with diagnosed students or other factors.

Beyond the discrepancy compared to lifetime and current prevalence estimates, substantial individual variability in teachers’ comorbidity estimates (0–88%) was observed. Notably, despite this range, the overall mean estimate (36.56%) closely aligned with current epidemiological data, suggesting that while individual estimates varied, they were influenced by meaningful factors rather than random error. As discussed below, this variability was systematically related to predictors such as personal experience, current teaching exposure, and disorder-specific knowledge, suggesting that individual differences in background and professional context shape how educators perceive ASD+ADHD prevalence.

As predicted, teachers with personal experience (through self/family diagnoses) and those currently teaching students with ASD+ADHD provided higher prevalence estimates. This finding aligns with prior research suggesting that personal exposure to a condition can increase perceived prevalence due to availability bias—the tendency for individuals to judge the frequency of an event based on how easily they can recall instances of it [53]. This pattern suggests that firsthand exposure to ASD+ADHD may increase awareness of its co-occurrence, whereas those without direct experience may be less attuned to its prevalence. Future studies should examine whether the breadth of exposure, such as the number of comorbid students taught over time, affects estimates differently than simply having had some degree of experience.

In contrast, total years of teaching experience was negatively related to prevalence estimates, contradicting our hypothesis that longer careers would also lead to higher estimates. One explanation is that general teaching tenure does not necessarily translate to increased exposure to ASD+ADHD, particularly if teachers have worked with different student populations throughout their careers. Rather than cumulative teaching experience shaping estimates, specific interactions with neurodiverse students may be more influential. Future studies could explore this longitudinally, tracking how estimates shift with increasing experience with ASD+ADHD students rather than simply accumulating years in the profession.

Total years of teaching experience was also strongly correlated with age (*r* = 0.726, *p <* 0.001), indicating that older teachers generally had more experience. An alternative explanation for the negative relationship between teaching tenure and prevalence estimates is that teachers with more experience may have completed their education and training at a time when ASD+ADHD comorbidity was less emphasized in educational and clinical discourse. Given that comorbid diagnoses have only been permitted since the DSM-5 2013 [6], teachers trained prior to this shift may be less likely to conceptualize ASD and ADHD as frequently co-occurring, potentially influencing the lower prevalence estimates. Future studies should inquire about the timing of education and training to explore this possibility.

While we did not make a priori predictions regarding the direction of disorder-specific knowledge effects, the findings revealed a contrasting pattern: greater ASD knowledge was associated with lower prevalence estimates, while ADHD knowledge was not a significant predictor. The negative association between ASD knowledge and prevalence estimates suggests that teachers with stronger ASD knowledge may conceptualize ASD and ADHD as distinct conditions rather than frequently co-occurring diagnoses. This pattern aligns with research on diagnostic overshadowing, where individuals with greater familiarity with one disorder may focus on its defining characteristics while overlooking its symptom overlap with another condition [54,55]. Alternatively, this could indicate underlying misconceptions about comorbidity [56], in which teachers with higher ASD knowledge are more likely to perceive ADHD symptoms as separate rather than part of a co-occurring profile. While ASD and ADHD frequently co-occur, the neurobiological basis of their comorbidity remains a subject of debate even in the research literature [57]. Some scholars argue that ASD and ADHD share overlapping neurodevelopmental pathways, while others suggest they remain distinct but comorbid conditions or even subtypes within a broader neurodevelopmental spectrum, e.g., [58,59]. If teachers conceptualize ASD and ADHD in rigid categorical terms, they may underestimate the extent to which symptoms can co-occur. Future studies should examine whether ASD-specific training reinforces these categorical distinctions and whether modifying training content could improve educators’ understanding of symptom overlap and its implications for classroom support.

Despite these conceptual challenges, prior training in ASD, ADHD, or ASD+ADHD was not significantly associated with teachers’ estimates of comorbidity prevalence. This suggests that standard professional development programs may not explicitly address the epidemiology of comorbid conditions, instead focusing more on intervention strategies for students already diagnosed, e.g., [60]. However, given that teachers often play a critical role in early identification and referrals [17,18,19], a lack of awareness of ASD+ADHD comorbidity could hinder timely support. Additional research could examine the content of teacher training programs to determine whether information on comorbidity is included. If so, modifying training content to highlight comorbid presentations could improve teachers’ understanding and recognition of ASD+ADHD.

While the present study identified key individual predictors of prevalence estimates, broader contextual factors may further contribute to this variability. Geographic diagnostic trends, school policies, and access to neurodevelopmental services likely influence differences in reported prevalence. For example, school districts with proactive screening and special education support systems may report higher identification rates, whereas regions with limited access to diagnostic specialists may have lower reported prevalence due to underdiagnosis [61,62]. In addition, disparities in professional development opportunities, funding allocations, and special education referral practices across schools and districts may shape teachers’ awareness of neurodevelopmental disorders and comorbidity [62,63]. Future research should further examine whether teachers’ estimates vary systematically by region, school policy structures, and access to neurodevelopmental support services, particularly in relation to screening and special education referral processes. Investigating systemic influences would provide a more comprehensive understanding of the factors shaping teachers’ perceptions of comorbidity beyond direct personal and professional experiences.

### 4.2. Perceived Preparedness

As predicted, teachers reported feeling less prepared to teach students with comorbid ASD+ADHD compared to those with only ASD or only ADHD. This finding reflects the added complexity of comorbid conditions and the challenge of applying strategies likely designed for single diagnoses. Several predictors emerged in alignment with our hypotheses: teaching self-efficacy, neurodiversity attitudes, and formal training were all positively associated with preparedness across all models.

Teaching self-efficacy was the strongest predictor across all models, consistent with research linking greater confidence to improved classroom adaptability [64] and effective inclusive education practices [65]. This finding aligns with Bandura’s self-efficacy theory [32], highlighting its role in shaping self-reported preparedness. Teachers with higher self-efficacy may feel more capable of adapting instructional strategies, implementing accommodations, and managing neurodiverse classrooms. Similarly, more positive neurodiversity attitudes aligned with higher preparedness, supporting prior findings that teacher perceptions shape inclusive practices [35,36]. Finally, training in ASD, ADHD, or ASD+ADHD significantly predicted preparedness, reinforcing the role of professional development in equipping educators to support neurodivergent students [66]. As inclusive education expands, structured training remains essential for fostering teacher confidence and effectiveness [67].

Several findings did not align with our initial predictions, highlighting complexities in how experience and knowledge shape preparedness. Despite having an influence on prevalence estimates, personal experience with ASD or ADHD did not predict preparedness in any model. While personal exposure may increase awareness [53], it may not translate into professional readiness to support students with neurodivergent conditions. Future research could explore whether certain types of personal experience (e.g., self-diagnosis, parenting, having a neurodivergent sibling) impact preparedness differently. Similarly, total years of teaching experience was not a significant predictor of preparedness for ASD, ADHD, or ASD+ADHD. This suggests that time in the profession alone does not necessarily enhance confidence in supporting neurodivergent students. One possibility is that newer teachers, who trained more recently, may have received stronger neurodiversity education, contributing to greater comfort with ASD students. Research also suggests that younger educators may hold more inclusive attitudes [68], which could further explain why preparedness for ASD was negatively related to years of teaching. Future studies should further explore whether shifts in teacher training have influenced generational differences in preparedness.

One key factor that did predict preparedness as expected was direct classroom experience with ASD+ADHD students. Teachers who currently instruct students with comorbid ASD+ADHD reported significantly higher preparedness for these students, but this effect did not extend to ASD or ADHD alone. This again reiterates the importance of hands-on exposure in developing confidence, suggesting that preparedness is shaped more by direct interaction than by overall teaching tenure. Teachers develop confidence based on direct classroom experience, and those who have actively worked with comorbid ASD+ADHD students may have adapted their teaching strategies accordingly. Future research should explore programs [69] to simulate this experience for teachers who have not worked with comorbid students.

Neither ASD-specific nor ADHD-specific knowledge significantly predicted preparedness to teach students with comorbid ASD+ADHD, suggesting that understanding each disorder in isolation may not translate to confidence in managing their combined challenges. Also, contrary to expectations, neither ASD knowledge predicted preparedness to teach ASD, nor did ADHD knowledge predict preparedness to teach ADHD. These findings indicate that disorder-specific knowledge alone may not be sufficient for fostering teaching confidence, aligning with prior research suggesting that training does not always translate to classroom preparedness [27,28]. However, greater ASD knowledge was negatively associated with preparedness to teach ADHD students. Teachers with stronger ASD knowledge may conceptualize ASD and ADHD as distinct rather than overlapping [57], reinforcing categorical thinking and limiting confidence in supporting ADHD students. This specialization effect may also explain why disorder-specific knowledge did not predict preparedness for comorbid ASD+ADHD. However, additional research would be needed to explore whether ASD-focused training inadvertently reduces perceived competence in managing ADHD-related behaviors. If so, professional development should emphasize cross-disorder strategies [38] that apply to both conditions.

### 4.3. Limitations and Future Directions

This present study is not without limitations. First, data collection was conducted entirely online, which limits our ability to verify participants’ current employment status or the specific diagnoses of students they teach. Although we took multiple steps to confirm eligibility, such as requiring participants to report their primary teaching role, grade levels taught, years of experience, and school type, self-report data inherently rely on participant accuracy. Future research should aim to replicate these findings using alternative recruitment strategies where employment and training credentials can be confirmed.

Further, while we recruited a nationwide sample through Prolific to enhance generalizability beyond a single school or state, our final sample was still predominantly white and identified as female. This demographic skew is likely reflective of both Prolific’s user base and broader trends in the Pre-K-12 teacher demographics in the U.S. [70]. Additionally, although the sample size was sufficient for our analyses, future research with larger and more demographically diverse teacher populations would strengthen generalizability and allow for a more detailed examination of subgroup differences. Future studies should prioritize diverse recruitment to better understand whether these findings generalize across educators from different racial, ethnic, and socioeconomic backgrounds.

The present analyses focused on teachers’ current experiences with ASD+ADHD students. To minimize recall bias, we excluded a small sample (*n* = 11) of participants who had taught ASD+ADHD students in the past but were not currently doing so. However, future studies using a lifetime exposure measure may provide a more comprehensive understanding of how past experiences shape teachers’ perceptions and preparedness.

Additionally, one of the strongest predictors (i.e., prior training) of both prevalence estimates and preparedness was measured categorically. While teachers described the types of training they received (e.g., formal workshops, coursework), all responses were collapsed into a single categorical variable, potentially obscuring meaningful differences in training content or intensity. Future studies should consider assessing training hours, specific instructional components, and academic qualifications to better capture the impact of professional development on teacher preparedness. For example, investigating whether teachers with formal degrees or coursework in special education or neurodiversity feel more prepared could provide further insight into how academic training shapes confidence and instructional effectiveness.

Given the wide range of prevalence estimates observed, future studies may benefit from subgroup analyses comparing newer vs. more experienced teachers and those from different educational settings (e.g., general education vs. special education classrooms). Further, incorporating standardized criteria for prevalence estimation may improve consistency in reporting and understanding how knowledge and exposure shape educators’ perceptions of ASD+ADHD co-occurrence.

Finally, while this study accounted for individual differences in training, experience, and disorder-specific knowledge, broader systemic factors may also play a role. These include school policies, geographic diagnostic rates, and variations in teacher training programs. However, these factors were not directly assessed in this study. Future research should examine how additional differences across educational settings influence teachers’ awareness of ASD+ADHD, particularly in relation to diagnostic access, screening practices, and referral pathways.

## 5. Conclusions

The present study provides novel insights into how teachers perceive ASD+ADHD comorbidity and their preparedness to instruct students with co-occurring diagnoses. Findings suggest that direct experience and formal training influence both awareness and preparedness, yet disorder-specific knowledge alone does not necessarily translate into confidence in teaching students with comorbid diagnoses. The lack of association between years of teaching experience and preparedness further underscores the need for targeted professional development that explicitly addresses ASD+ADHD as an integrated condition. As inclusive education continues to evolve, ensuring teachers are equipped to meet the diverse needs of their students will require a greater emphasis on comorbidity-focused training. Future research should evaluate the effectiveness of cross-condition training strategies and explore how teacher preparedness impacts classroom practices and student outcomes.

## Figures and Tables

**Table 1:** Teaching-related characteristics of participants (*N* = 199).

Variable	n	%
**Primary Role**		
General Education/Classroom Teacher	131	65.8%
Special Education	26	13.1%
Gifted Education	1	0.5%
Support Teacher/Teacher’s Aide	15	7.5%
Specialist (e.g., Speech, ELL)	17	8.5%
Special Topics (e.g., music, PE, tech)	7	3.5%
Administration	2	1.0%
**Teaching (Grade) Level ^1^**		
Early Education (Pre-K/K, ages 3–5)	37	18.6%
Elementary (1st–5th, ages 6–10)	57	28.6%
Elementary (1st–5th, ages 11–13)	42	21.1%
Elementary (1st–5th, ages 14–18)	63	31.7%
**School Type ^2^**		
Public (Non-Charter)	147	73.9%
Public (Charter)	21	10.6%
Private (Religious)	17	8.5%
Private (Non-Religious)	11	5.5%
Alternative	3	1.5%
**School Setting**		
Urban	56	28.1%
Suburban	104	52.3%
Rural	36	18.1%
Unsure	3	1.5%
**Student SES ^3^**		
Primarily Low-Income	87	43.7%
Primarily Middle-Income	59	29.6%
Primarily High-Income	16	8.0%
Diverse-Mixed	37	18.6%
**Integration ^4^**		
Fully Integrated (General Education)	114	57.3%
Separate (Special Education Only)	9	4.5%
Mixed (Combination of Settings)	76	38.2%

^1^ For Teaching Level, teachers who reported experience across multiple grade levels were categorized based on the lowest grade level they currently teach. Public (Charter) refers to publicly funded but independently operated schools. ^2^ Private (Religious) schools include faith-based institutions, whereas Private (Non-Religious) refers to secular private schools. Alternative Schools serve students with unique educational needs. ^3^ SES represents the general socioeconomic background of the majority of the students at the school. ^4^ Integration represents whether students with diagnoses are or are not integrated into general classrooms.

**Table 2:** Regression predicting teachers’ prevalence estimates of co-occurring ASD+ADHD.

Predictor	*B*	*SE B*	*β*
**Background Factors**			
Total Years Teaching	−0.387	0.165	−0.161 *
Personal Experience ^1^	5.886	2.947	0.141 *
**Exposure and Experience**			
Currently Teaching ASD+ADHD ^2^	9.851	2.864	0.238 ***
Training Received ^3^	−0.613	2.822	−0.015
**Knowledge and Attitudes**			
ASD Knowledge (PAK-M) ^4^	−0.322	0.132	−0.225 *
ADHD Knowledge (SASK) ^5^	−0.013	0.231	−0.005
Neurodiversity Attitudes (NDAQ) ^6^	0.184	0.113	0.136
**Model Summary**			
*R*^2^ (Adj.)	0.108		
*F* for Δ*R*^2^	4.419 **		

* *p* < 0.05, ** *p* < 0.01, *** *p* < 0.001. ^1^ Personal Experience represents self and/or family member diagnosed with any ASD, ADHD or both (0 = No, 1 = Yes). ^2^ Currently comorbid ASD+ADHD Teaching (0 = No, 1 = Yes). ^3^ Training Received = any formal training related to ASD, ADHD, or comorbid ASD+ADHD (0 = No, 1 = Yes). ^4^ PAK-M = Participatory Autism Knowledge Measure PAK-M. ^5^ SASK = Scale of ADHD-Specific Knowledge. ^6^ NDAQ = Neurodiversity Attitudes = Neurodiversity Attitudes Questionnaire.

**Table 3:** Regression predicting teachers preparedness to teach students with co-occurring ASD+ADHD.

Predictor	*B*	*SE B*	*β*
**Background Factors**			
Total Years Teaching	0.014	0.008	0.107
Personal Experience ^1^	−0.017	0.137	−0.008
**Exposure and Experience**			
Currently Teaching ASD+ADHD ^2^	0.556	0.133	0.256 ***
Training Received ^3^	0.399	0.132	0.184 **
**Knowledge and Attitudes**			
ASD Knowledge (PAK-M) ^4^	−0.011	0.006	−0.146
ADHD Knowledge (SASK) ^5^	−0.008	0.011	−0.054
Neurodiversity Attitudes (NDAQ) ^6^	0.018	0.005	0.251 **
Teaching Self-Efficacy (TSES) ^7^	0.028	0.006	0.316 ***
**Model Summary**			
*R*^2^ (Adj.)	0.303		
*F* for Δ*R*^2^	11.738 ***		

** *p* < 0.01, *** *p* < 0.001. ^1^ Personal Experience represents self and/or family member diagnosed with any ASD, ADHD, or both (0 = No, 1 = Yes). ^2^ Currently comorbid ASD+ADHD Teaching (0 = No, 1 = Yes). ^3^ Training Received = any formal training related to ASD, ADHD, or comorbid ASD+ADHD (0 = No, 1 = Yes). ^4^ PAK-M = Participatory Autism Knowledge Measure PAK-M. ^5^ SASK = Scale of ADHD-Specific Knowledge. ^6^ NDAQ = Neurodiversity Attitudes = Neurodiversity Attitudes Questionnaire. ^7^ TSES = Teacher Sense of Self Efficacy Scale.

## Data Availability

The original contributions presented in this study are included in the article/Supplementary Materials. Further inquiries can be directed to the corresponding author.

## Supplementary Materials

The following supporting information can be downloaded at https://www.mdpi.com/article/10.3390/children12030342/s1: Table S1: Regression predicting teachers preparedness to teach students with ASD; Table S2: Regression predicting teachers preparedness to teach students with ADHD.

## Abbreviations

The following abbreviations are used in this manuscript:

ASD	Autism Spectrum Disorder
ADHD	Attention-Deficit/Hyperactivity Disorder
ASD+ADHD	Co-occurring (comorbid) Autism Spectrum Disorder and Attention Deficit/Hyperactivity Disorder
PAK-M	Participatory Autism Knowledge Measure
SASK	Scale of ADHD-Specific Knowledge
NDAQ	Neurodiversity Attitudes Questionnaire
TSES	Teacher Sense of Self Efficacy Scale
