# Supplementary material for: Pre-K–12 Teachers’ Views on ASD+ADHD: Prevalence Estimates and Teaching Preparedness
Source: Children (Basel). 2025 Mar 9;12(3):342. doi: 10.3390/children12030342 (PMC11941484; doi:10.3390/children12030342)
Supplement: Supplementary file 1 [file children-12-00342-s001.zip › children-3495521-supplementary.pdf]

Supplementary Table S1. Regression Predicting Teachers Preparedness to Teach Students with ASD.

Predictor	*B*	*SE B*	β
**Background Factors**			
Total Years Teaching	0.009	0.007	0.082
Personal Experience[1]	0.032	0.127	0.016
**Exposure and Experience**			
Currently Teaching ASD+ADHD[2]	0.201	0.123	0.103
Training Received[3]	0.524	0.122	0.270***
**Knowledge and Attitudes**			
ASD Knowledge (PAK-M)[4]	-0.001	0.006	-0.016
ADHD Knowledge (SASK)[5]	-0.016	0.010	-0.131
Neurodiversity Attitudes (NDAQ)[6]	0.019	0.005	0.298***
Teaching Self-Efficacy (TSES)[7]	0.017	0.005	0.215**
**Model Summary**			
R^2 (Adj.)	0.254		
F for ΔR^2	9.414***		

** $p < 0.01$, *** $p < 0.001$. ** p < 0.01, *** p < 0.001. [1]Personal Experience represents self and/or family member diagnosed with any ASD, ADHD, or both (0 = No, 1 = Yes). [2]Currently comorbid ASD+ADHD Teaching (0 = No, 1 = Yes). [3]Training Received = any formal training related to ASD, ADHD, or comorbid ASD+ADHD (0 = No, 1 = Yes). [4]PAK-M = Participatory Autism Knowledge Measure PAK-M. [5]SASK = Scale of ADHD-Specific Knowledge. [6]NDAQ = Neurodiversity Attitudes = Neurodiversity Attitudes Questionnaire. [7]TSES = Teacher Sense of Self Efficacy Scale.

Supplementary Table S2. Regression Predicting Teachers Preparedness to Teach Students with ADHD.

Predictor	*B*	*SE B*	β
**Background Factors**			
Total Years Teaching	0.013	0.007	0.124
Personal Experience[1]	0.186	0.117	0.105
**Exposure and Experience**			
Currently Teaching ASD+ADHD[2]	0.043	0.113	0.024
Training Received[3]	0.261	0.112	0.149*
**Knowledge and Attitudes**			
ASD Knowledge (PAK-M)[4]	-0.012	0.005	-0.202*
ADHD Knowledge (SASK)[5]	0.001	0.009	0.007
Neurodiversity Attitudes (NDAQ)[6]	0.013	0.005	0.229**
Teaching Self-Efficacy (TSES)[7]	0.024	0.005	0.335***
**Model Summary**			
R^2 (Adj.)	0.226		
F for ΔR^2	8.246***		

* $p < 0.05$, ** $p < 0.01$, *** $p < 0.001$. [1]Personal Experience represents self and/or family member diagnosed with any ASD, ADHD, or both (0 = No, 1 = Yes). [2]Currently comorbid ASD+ADHD Teaching (0 = No, 1 = Yes). [3]Training Received = any formal training related to ASD, ADHD, or comorbid ASD+ADHD (0 = No, 1 = Yes). [4]PAK-M = Participatory Autism Knowledge Measure PAK-M. [5]SASK = Scale of ADHD-Specific Knowledge. [6]NDAQ = Neurodiversity Attitudes = Neurodiversity Attitudes Questionnaire. [7]TSES = Teacher Sense of Self Efficacy Scale.
